# Modeling the effect of grazing on carbon and water use efficiencies in grasslands on the Qinghai–Tibet Plateau

**DOI:** 10.1186/s12862-024-02215-4

**Published:** 2024-02-27

**Authors:** Xiaotao Huang, Geping Luo, Zhen Ma, Buqing Yao, Yangong Du, Yongsheng Yang

**Affiliations:** 1 School of Geographical Sciences and Tourism, Zhaotong University, 657000 Zhaotong, Yunnan China; 2 State Key Laboratory of Desert and Oasis Ecology, Xinjiang Institute of Ecology and Geography, Chinese Academy of Sciences, 830011 Urumqi, Xinjiang China; 3 Key Laboratory of Restoration Ecology for Cold Regions Laboratory in Qinghai, Northwest Institute of Plateau Biology, Chinese Academy of Sciences, 810008 Xining, Qinghai China

**Keywords:** CUE, WUE, Spatiotemporal dynamics, Grazing, Model, Qinghai–Tibet Plateau

## Abstract

**Background:**

Carbon and water use efficiencies (CUE and WUE, respectively) are vital indicators of the adaptability of plants to environmental conditions. However, the effects of grazing and climate change on the spatiotemporal changes in CUE and WUE in Qinghai–Tibet Plateau grasslands (QTPG) are still unclear.

**Results:**

Using the enhanced Biome-BGCMuSo model in combination with observed data, we estimated and analyzed the spatiotemporal variations in CUE and WUE and their responses to grazing in QTPG from 1979 to 2018. The mean annual CUE was 0.7066 in QTPG from 1979 to 2018 under the actual climate scenario. In general, the grassland CUE was low in the southeast and high in the northwest. Grazing generally decreased CUE in QTPG from 1979 to 2018, and there was an increasing trend in the difference in CUE between the grazing and nongrazing scenarios. The difference in CUE was generally greater in the northwest than in the southeast. The mean annual WUE was 0.5591 g C/kg H_2_O in QTPG from 1979 to 2018 under the actual climate scenario. After 2000, the grassland WUE exhibited a fluctuating upward trend. In general, the grassland WUE was greater in the southeast than in the northwest. Grazing generally decreased WUE in QTPG from 1979 to 2018, and there was an increasing trend in the difference in WUE between the grazing and nongrazing scenarios. The difference in WUE was generally greater in the northwest than in the southeast.

**Conclusions:**

The findings of this study suggested that the spatiotemporal changes in CUE and WUE in QTPG were closely related to changes in the natural environment and grazing management.

## Introduction

Carbon use efficiency (CUE) is defined as the proportion of carbon utilized for growth relative to the total carbon absorbed by vegetation [1]. It is an important indicator for estimating the efficiency of the exchange between atmospheric CO_2_ and plant biomass. And it also indicates carbon allocation in storage and consumption by vegetation and can determine the impact of respiration on vegetation net productivity [2]. A high CUE indicates that autotrophic respiration is responsible for a small fraction of gross ecosystem productivity and that the ecosystem has high carbon sequestration efficiency [3]. Ecosystem WUE is usually defined as the amount of CO_2_ fixed or the dry matter produced by per unit of water consumption in the ecosystem [4]. It is a key index used to measure the degree of coupling between ecosystem water and carbon and reflects the relationship between water consumption and photosynthetic production in the ecosystem [5, 6]. A higher WUE indicates that more photosynthetic products can be obtained by using the available water resources. Both WUE and CUE are important gauges of the adaptability of plants to changes in environmental conditions such as anthropogenic interference and climate change [7, 8]. Understanding CUE and WUE is highly important for the study of vegetation growth, the carbon sequestration capacity of terrestrial ecosystems and even global change [9, 10]. In particular, determining the spatiotemporal changes in CUE and WUE is highly important for quantifying the response of terrestrial ecosystems to anthropogenic activities and climate change [11, 12].

Grasslands, which are widely distributed on Earth’s surface, play an important role in maintaining global and regional ecological balance [13–15]. Moreover, grassland ecosystems are sensitive to anthropogenic activities and climate change [16–18]. However, we haven’t found any systematic reports on the effects of grazing on CUE over large areas in previous publications. Moreover, we haven’t found any systematic reports on the effects of grazing on WUE over large areas in alpine grasslands in previous publications.

The Qinghai–Tibet Plateau (QTP) is known as “the roof of the world” and the “Asian water tower” because it is the highest plateau on Earth and the birthplace of some large rivers [19, 20]. The region has a unique plateau climate and is very sensitive to global change. Moreover, the QTP is an ecological barrier in Asia and an ideal site for studying the response of terrestrial ecosystems to global change. In addition, the QTP plays an important role in regulating the regional and global carbon balance [8, 21]. Thus, studying ecosystem CUE and WUE on the QTP is highly important for understanding the carbon sequestration capacity and mechanisms of the carbon and water cycles of terrestrial ecosystems and further protecting the ecological environment. The alpine grassland ecosystem is the dominant ecosystem type on the QTP, accounting for approximately 60% of the total area [20, 22]. The susceptibility of alpine grasslands to interference makes the ecology in this region extremely sensitive to anthropogenic activities and climate change [23]. The complexity of the carbon and water cycles in grasslands of the Qinghai–Tibet Plateau (QTPG) has significantly increased due to climate warming, extreme climate events and intensified human activities (e.g., grazing) [8, 23]. Alpine grasslands have experienced extensive degradation in this region, mainly due to unsustainable grazing [8, 17]. Grassland degradation has led to a low and unstable level of animal husbandry and has severely restricted the survival of local people. It has also severely affected sustainable development in the middle and lower reaches of large rivers and Southeast Asian countries [20, 23]. However, there are no systematic reports on the spatiotemporal changes in CUE and its response to grazing on the QTPG [8]. W Liu, X Mo, S Liu, Z Lin and C Lv [23] investigated the spatiotemporal variations in WUE in alpine grasslands on the QTP during 2001–2017 using the VIP distributed ecohydrological model. N Ma and Y Zhang [24] also investigated the change in WUE of alpine grasslands on the QTP between 1982 and 2016 using a coupled carbon–water model. However, the results from these two studies differed greatly. In addition, the effect of grazing on the spatiotemporal changes in WUE in QTPG has not been reported. The lack of knowledge about CUE and WUE is inconducive to the sustainable use of local grassland resources.

The establishment of terrestrial ecosystem carbon–water cycle models has increasingly shown their advantages in in-depth analysis of the spatiotemporal characteristics of ecosystem carbon–water cycles and their responses to anthropogenic activities and climate change. This approach has become the main research method in this field [25]. Many related models have been established. Examples include Biome-BGC (Biome-BioGeoChemical), TEM (Terrestrial Ecosystem Model), CENTURY, DNDC (DeNitrification DeComposition), DLEM (Dynamic Land Ecosystem Model), and IBIS (the Integrated Biosphere Simulator). Among these established models, the Biome-BGC model has been successfully used for studying the carbon and water functions of ecosystems in different regions (including grasslands on the QTP) [26, 27]. D Hidy, Z Barcza, H Marjanovic, MZO Sever, L Dobor, G Gelybo, N Fodor, K Pinter, G Churkina, S Running, et al. [28] constructed Biome-BGCMuSo, further improving the structure of Biome-BGC. Previous studies showed that, compared with Biome-BGC, Biome-BGCMuSo performed better at simulating carbon and water fluxes and storages in terrestrial ecosystems. In particular, Biome-BGCMuSo can more accurately simulate the impact of several special physiological and ecological processes caused by energy and water stress on vegetation growth [28]. Vegetation growth on the QTP is generally limited by energy availability because of high elevation and low temperature. Arid and semiarid grasslands are widely distributed in this region [8, 19]. The points mentioned above suggest that the Biome-BGCMuSo model can assess the carbon and water functions of QTPG more accurately than the Biome-BGC model. Moreover, grazing is represented in Biome-BGCMuSo [28].

The aim of this study was to simulate and analyze (1) the spatiotemporal changes in CUE and WUE and (2) their responses to grazing in QTPG from 1979 to 2018 based on the Biome-BGCMuSo model.

## Methods and materials

### Study area

**Fig. 1:**
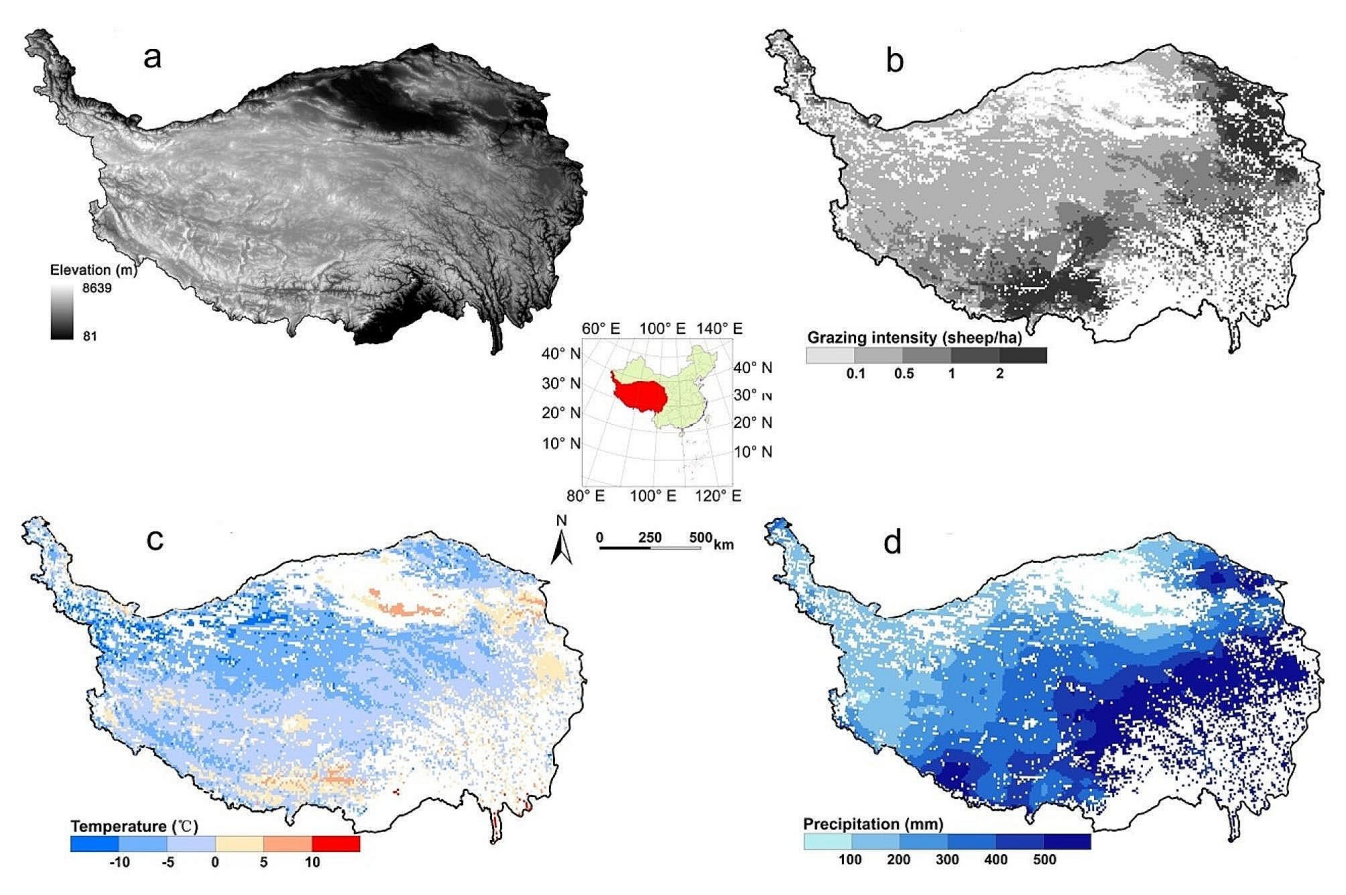
Distribution of the elevation (**a**), yearly mean grazing intensity (**b**), yearly mean temperature (**c**), and yearly mean rainfall (**d**) in grasslands of the Qinghai-Tibetan Plateau from 1979 to 2018

The QTP (73°18′-104°47′E, 26°00′-39°47′N) is located in southwestern China. It is the plateau with the highest elevation on Earth and the largest area in China. The average elevation exceeds 4000 m, and the area is 2.57 × 10^6^ km^2^. This plateau is the birthplace of several major rivers (e.g., Yellow River, Yangtze River, Nujiang River, Lancang River, and Yarlung Zangbo River) in Asia. The ecological environment has a great impact on the lower and middle reaches of the rivers and even the whole Northern Hemisphere [20, 23]. The average annual temperature is low, ranging from − 15 °C to 10 °C. The temperature decreases from the southeast to the northwest. The average annual precipitation is approximately 400 mm. The precipitation is unevenly distributed, decreases from the southeast to the northwest and is mainly concentrated from June to September. The alpine grassland ecosystem is the main ecosystem type in this region, accounting for approximately 60% of the total area. It fulfills an important role in maintaining regional ecological balance and regulating climate. The grasslands in this region are also an important animal husbandry production base in China and an important source of support for local herdsmen. However, grassland degradation in the area was widespread mainly due to overgrazing (Fig. 1) [20, 23].

### Method

Biome-BGCMuSo is a process-based model adapted from the commonly used Biome-BGC model to increase the ability to simulate the water and carbon cycles in terrestrial ecosystems. Compared with the old Biome-BGC model, some model structures were improved in the Biome-BGCMuSo model. For example, a ten-layer soil submodel was implemented, plant senescence related to drought was included, vegetation phenology was improved, and management modules were developed (e.g., to represent grazing). A detailed description of the improvements can be found in the publication by D Hidy, Z Barcza, H Marjanovic, MZO Sever, L Dobor, G Gelybo, N Fodor, K Pinter, G Churkina, S Running, et al. [28].

Gross primary productivity (GPP), the photosynthetic CO_2_ uptake, was estimated using Farquhar’s photosynthesis routine and the enzyme kinetics model in the Biome-BGCMuSo model [28–30]. Autotrophic respiration is equal to the sum of maintenance respiration and growth respiration. Maintenance respiration is calculated as a function of the temperature and the nitrogen content of living material [28]. Growth respiration is calculated as the proportion of carbon allocated to the different plant compartments [28].

Net primary productivity (NPP) is the difference between GPP and autotrophic respiration. Evapotranspiration (ET) is calculated based on the Penman–Monteith method [28].

In this study, grassland CUE was calculated as follows:

1 $$ \text{C}\text{U}\text{E} = \text{N}\text{P}\text{P}/\text{G}\text{P}\text{P}$$

Grassland WUE was calculated as follows:

2 $$ \text{W}\text{U}\text{E} = \text{G}\text{P}\text{P}/\text{E}\text{T}$$

In this study, two scenarios (actual climate scenario with no grazing and actual grazing scenario) were designed to study the impacts of grazing on CUE and WUE. The changes between the two scenarios were used to identify the effect of grazing on CUE and WUE.

When running the Biome-BGCMuSo model in this study, five input files were used. The initialization file (first file) included the physical and climatic characteristics, the time frame for the simulation, and variable lists for storage in output files, etc. The meteorological data file (second file) contained daily values for the required meteorological data (e.g., air temperature, precipitation). In the Biome-BGCMuSo model, all years were assumed to be 365 days, so we dropped 31 December from leap years. The ecophysiological constants file (third file) contained the parameters of the vegetation (e.g., maximum stomatal conductance, ratio of leaf C:N, and allocation ratios). The soil properties file (fourth file) contained detailed soil information (e.g., soil composition and characteristic soil water content). The management file (fifth file) allowed the simulation of grazing.

The modeling process includes three phases. In the first phase, a spin-up simulation is run to calculate initial state variable values. The model initiates with low soil nitrogen and carbon and continues until a steady state is reached with the climate. The second phase, transient simulation, is performed to prevent unwanted steep changes in the environmental state between the first phase and third phase. It starts with the endpoint of the regular spin-up and produces the inputs for the normal phase. The third phase, the normal simulation, is performed to produce the model outputs.

Biome-BGCMuSo was originally used for site simulation [28]. To identify large-scale CUE and WUE, the QTPG were assumed to be composed of grids with a resolution of 10 × 10 km, and the Biome-BGCMuSo model was run on the grids using the loop program in R. The outputs of the model were in the ASCII format. We converted the outputs from ASCII to raster format using R and Python programs. The spatially explicit results were subsequently displayed on a map.

### Model inputs

The model inputs included climate and grazing data and other ancillary data. All the inputs were extracted and smoothed to a 10 × 10 km resolution to facilitate the operation of the model in different grid cells using Python and R programs.

Climate data constitute the most important forcing data of the Biome-BGCMuSo model and include the daily maximum and minimum air temperature (°C), mean daytime air temperature (°C), daily rainfall (cm), mean daylight deficit in vapor pressure (Pa), daylight average shortwave radiation flux density (W/m^2^), and day length (s). All the data were sourced from the China Meteorological Forcing Dataset (CMFD) [31]. This dataset has a resolution of 10 × 10 km and a time range from January 1979 to December 2018. Previous studies have shown that the accuracy of these data is between that of observation data and remote sensing data and better than that of other existing reanalysis data compared with observation data (including observation data sampled on the QTP) [31].

The grazing data included intensity and timing. The grazing intensity data were derived from the webpage of the United Nations Food and Agriculture Organization (FAO) “Gridded Livestock of the World” (GLW) [32] and livestock statistics from local governments. The global distributions of livestock for 2010 can be downloaded from the GLW, a peer-reviewed spatial dataset with a spatial resolution of 5 arc minutes. The distributions of livestock for 2010 in QTPG were extracted from the GLW and corrected by comparison to livestock statistics for 2010 for different regions in QTPG from local governments. To obtain grazing intensity data for a time series from 1979 to 2018 and further ensure high accuracy of these data, these grazing intensity data were produced by linear interpolation using local government livestock statistics for different regions in QTPG from 1979 to 2018. The timing of grazing was set in accordance with a field survey of local herders. This study converted all livestock into sheep units according to the conversion coefficient from the Ministry of Agriculture and the field survey of local herders. One yak = 4.5 sheep, one cow = six sheep, one camel = eight sheep, one goat = 0.9 sheep, and one horse = six sheep.

Other ancillary data included soil data, physiological and ecological parameters, and site information. The soil data (including soil water content, soil composition, and soil pH) were extracted from the harmonized global soil database [33]. The physiological and ecological parameters (including the day of the year on which new growth began and the day of the year on which litterfall ended, C:N ratio of fine roots, C:N ratio of leaves, average specific leaf area in the canopy, allocation ratios, and maximum stomatal conductance) were derived from default parameterizations in the model and local surveys [34]. The site information included latitude, elevation, average annual air temperature, and the range of air temperatures. Site latitude and elevation data were extracted from the geospatial data cloud. The mean annual air temperature and mean annual air temperature range were extracted from the CMFD.

## Results

### Model validation

**Fig. 2:**
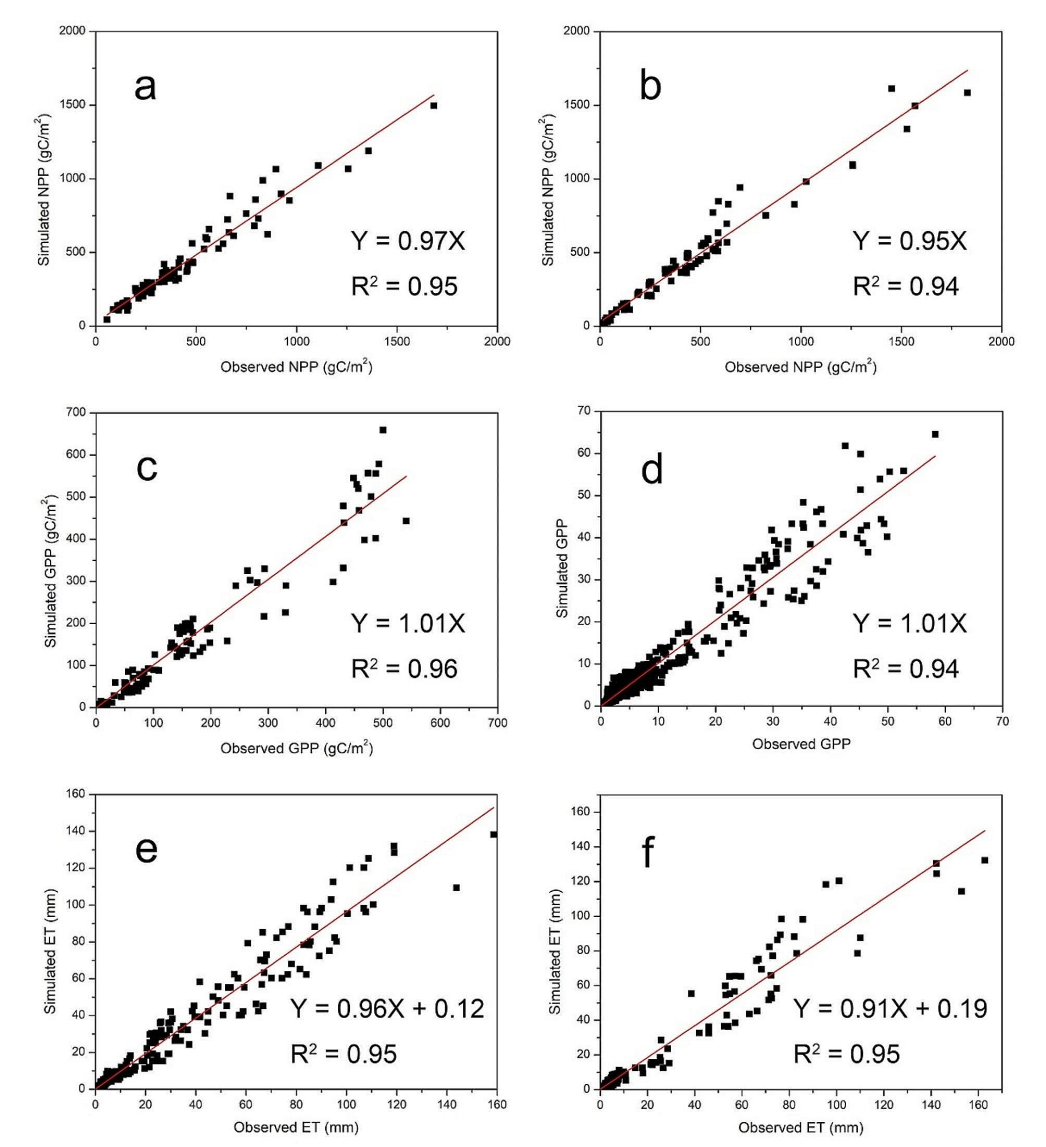
Comparison of modeled and observed NPP under grazing (**a**) and no grazing (**b**) conditions, GPP under grazing (**c**) and no grazing (**d**) conditions and ET under grazing (**e**) and no grazing (**f**) conditions (NPP–net primary productivity, GPP–gross primary productivity, ET–evapotranspiration).

Consistency between simulated results and observations is essential for establishing the credibility of model outputs. To ensure the reliability of the simulated results of the Biome-BGCMuSo model, we collected observed data (including NPP, GPP and ET) from the QTPG. These observed data were collected from either previous literature or field surveys [35–49]. In total, 184 NPP samples were distributed in 87 NPP plots, 1591 GPP samples were distributed in 10 GPP plots, and 675 ET samples were distributed in 17 ET plots. Among the NPP sampling plots, there were 39 plots (88 samples) with grazing and 48 plots (96 samples) without grazing. All these observed NPP data were converted from the annual peak biomass due to the high difficulty in directly observing NPP. The vegetation biomass was obtained by the standard harvesting method using a 0.5 m × 0.5 m quadrat with five replicates or more [40, 41]. Among the GPP sampling plots, there were 7 plots (622 samples) with grazing and 3 plots (969 samples) without grazing. All these observed GPP data were collected using the eddy covariance technique [35–39]. Among the ET sampling plots, there were 11 plots (265 samples) with grazing and 6 plots (410 samples) without grazing. These observed ET data were collected in 8 plots using the eddy covariance technique, 6 plots using a lysimeter, and 3 plots using a Bowen ratio tower [36, 39, 42–49]. The model outputs were validated by comparison with the observations, which revealed that the model performed well in terms of simulating NPP under both grazing (R^2^ = 0.95) and no grazing (R^2^ = 0.94) scenarios; GPP under both grazing (R^2^ = 0.96) and no grazing (R^2^ = 0.94) scenarios; and ET under both grazing (R^2^ = 0.95) and no grazing (R^2^ = 0.95) scenarios (Fig. 2).

### CUE and WUE

**Fig. 3:**
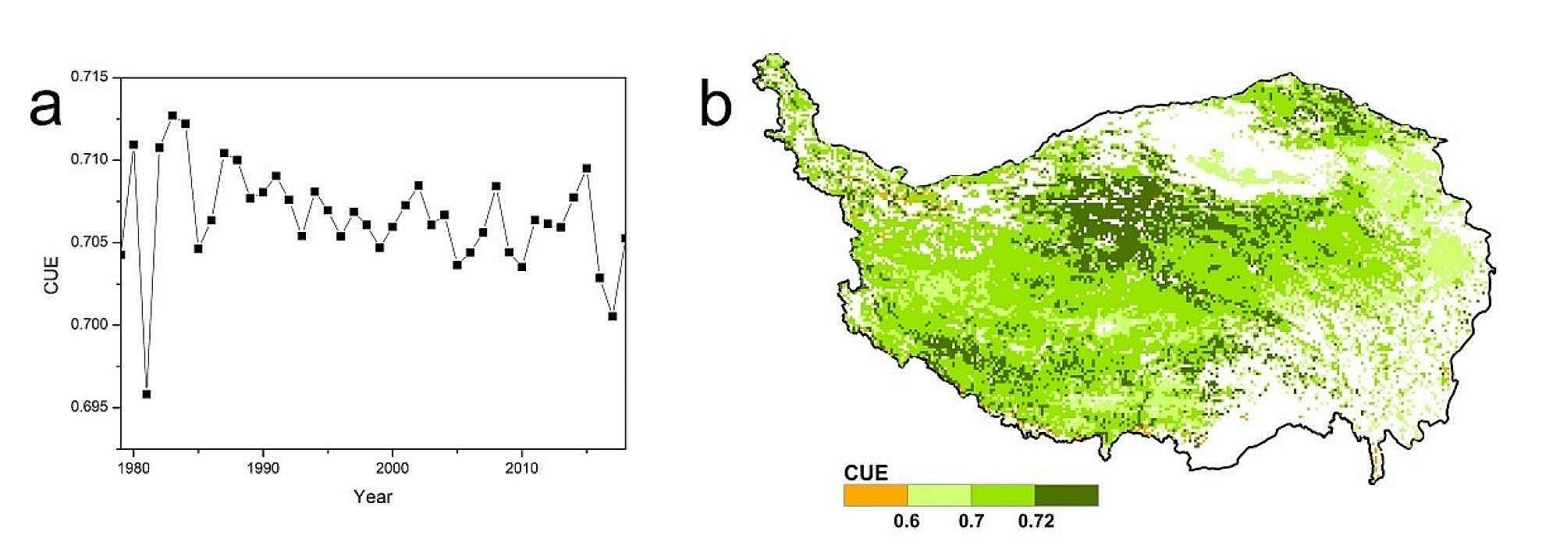
Temporal (**a**) and spatial (**b**) changes in CUE in grasslands of the Qinghai–Tibet Plateau from 1979 to 2018

The average annual CUE was 0.7066 in QTPG from 1979 to 2018 under the actual climate scenario. The annual values fluctuated between 0.6958 and 0.7127 and showed a weak fluctuating downward trend during this period. In general, the spatial heterogeneity of the average annual CUE was not large in QTPG from 1979 to 2018. The average annual CUE was between 0.6 and 0.72 in most regions, accounting for 80.00% of the total area. High CUE was predominantly found in the central and western QTP areas and the Qilian Mountains. Low values were predominantly found in the eastern and part of the southern regions (Fig. 3).

**Fig. 4:**
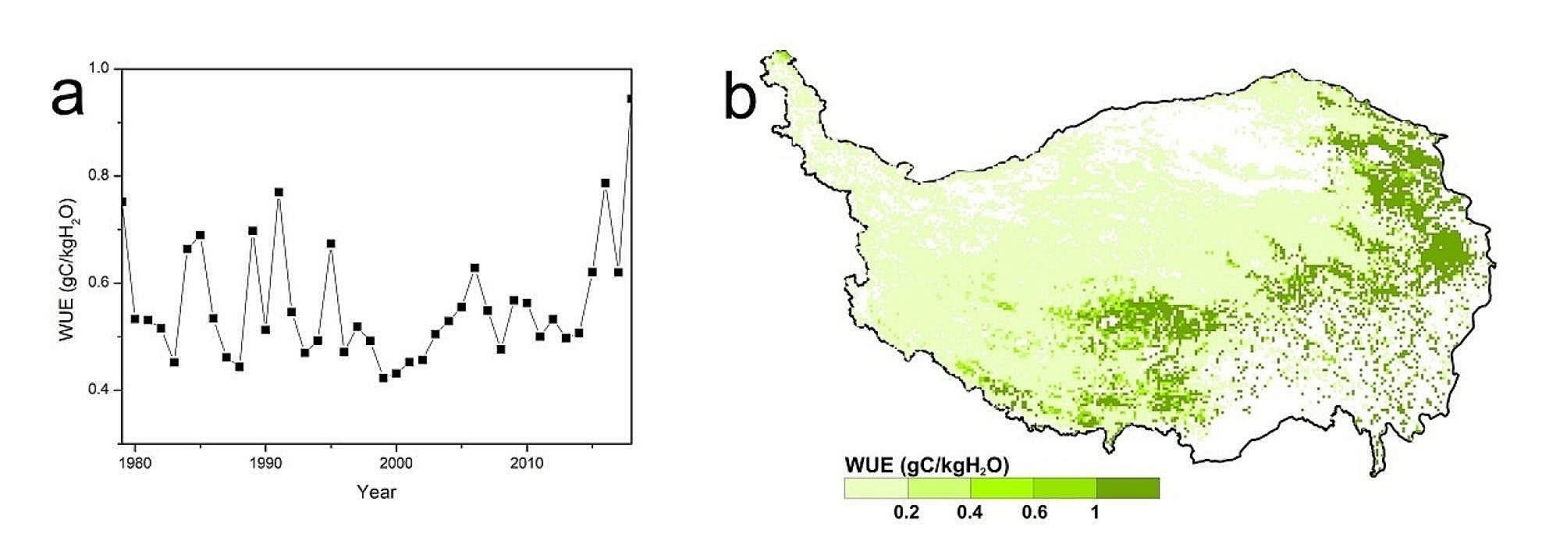
Temporal (**a**) and spatial (**b**) changes in WUE in grasslands of the Qinghai–Tibet Plateau from 1979 to 2018

The average annual WUE was 0.5591 gC/kgH_2_O in QTPG under the actual climate scenario from 1979 to 2018. The annual values varied between 0.4436 gC/kgH_2_O and 0.9442 gC/kgH_2_O. After 2000, the grassland WUE on the QTP exhibited a fluctuating upward trend, with a mean annual rate of 0.0273 gC/kgH_2_O. There was obvious spatial heterogeneity in the average annual WUE in QTPG from 1979 to 2018. The most widely observed average annual WUE was less than 0.2 gC/kgH_2_O (accounting for 76.45%), and this value was mainly concentrated in the northwest. The second most widely observed average annual WUE was greater than 1 (accounting for 17.93%), and this value was predominantly distributed in the southern and eastern parts of the QTPG (Fig. 4).

### Grazing effect on CUE and WUE

**Fig. 5:**
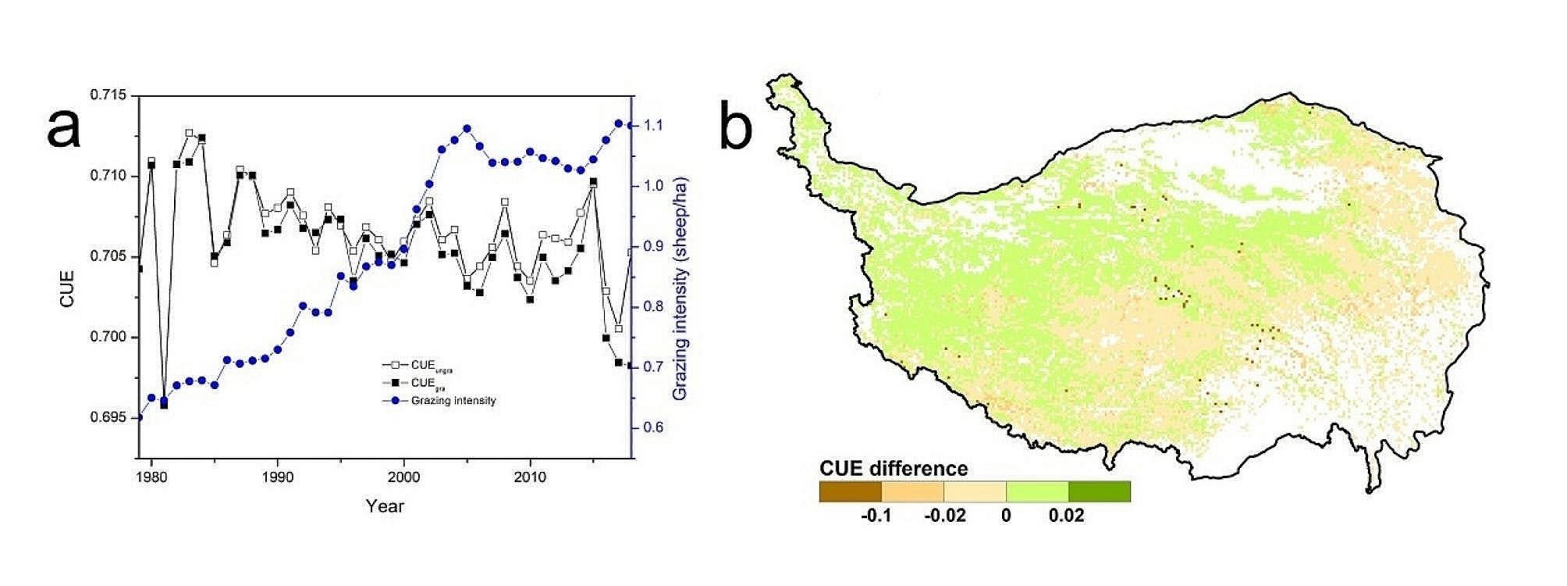
Impacts of grazing on temporal (**a**) and spatial (**b**) changes in CUE in grasslands of the Qinghai–Tibet Plateau from 1979 to 2018 (the difference in CUE represents the mean annual difference in CUE between the grazing and no grazing scenarios from 1979 to 2018)

Under the actual grazing scenario, the mean annual CUE was 0.7056 in QTPG from 1979 to 2018. Grazing generally decreased CUE, and the difference in CUE between the grazing and no grazing scenarios showed an increasing trend. Between 1979 and 2018, the mean annual difference in CUE was between − 0.02 and 0.02 in most regions, indicating that grazing had a weak effect on CUE in most regions of the QTPG. The regions where grazing decreased CUE accounted for 48.75% of the total area and were predominantly found in the east and south. The regions where grazing increased CUE accounted for 50.45% of the total area and were predominantly found in the northwest (Fig. 5).

**Fig. 6:**
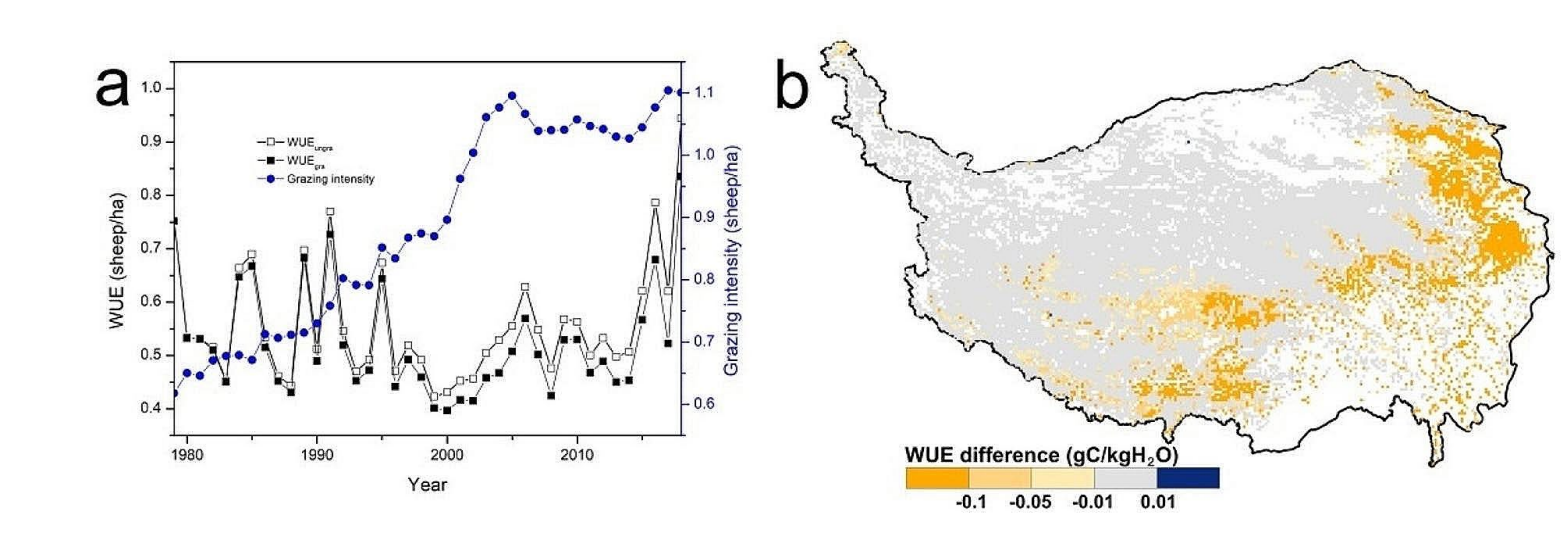
Impacts of grazing on temporal (**a**) and spatial (**b**) changes in WUE in grasslands of the Qinghai–Tibet Plateau from 1979 to 2018 (the difference in WUE represents the mean annual difference in WUE between the grazing and no grazing scenarios from 1979 to 2018)

The average annual WUE was 0.5237 gC/kgH_2_O in QTPG from 1979 to 2018 under the actual grazing scenario. Grazing generally decreased WUE, and the difference in WUE between the grazing and no grazing scenarios showed an increasing trend. From 1979 to 2018, grazing decreased WUE in most regions of the QTPG. The regions where grazing obviously decreased WUE (WUE difference < -0.1) accounted for 12.95% of the QTPG and were predominantly found in the east and south (Fig. 6).

## Discussion

### Spatiotemporal dynamics of CUE and WUE

In this study, we found that the spatiotemporal fluctuation in CUE was relatively weak in QTPG, and this finding was also supported by the results of previous studies by B Li, F Huang, L Qin, H Qi and N Sun [50]. The CUE in QTPG generally exhibited a fluctuating decreasing trend (Fig. 3a), which was closely related to global warming [2, 8]. Climate warming inevitably leads to an increase in the vegetation respiration rate, so the proportion of fixed carbon produced by plants declines. A greater percentage of CUE with low values occurred at relatively low elevations. More CUEs with high values occurred at relatively high elevations (Fig. 3b). This phenomenon was related to the temperature at different elevations; the temperature at low elevations was relatively high, resulting in a relatively high plant respiration rate and low CUE, while the opposite was true at high elevations [2, 8].

From 1979 to 2018, the average annual WUE was 0.5591 g C/kg H_2_O in QTPG. After 2000, the grassland WUE generally exhibited a fluctuating increasing trend, which was mainly due to the increase in GPP related to climate change [51, 52]. However, an average annual WUE less than 0.2 g C/kg H_2_O was the most widely distributed WUE, occurring mainly in the northwest. Due to the unsuitable climate and environmental conditions, the productivity of vegetation was very low in these regions, which led to low WUE. An average annual WUE greater than 1 g C/kg H_2_O was the second most widely distributed WUE, occurring mainly in the eastern and southern parts of the QTPG. The hydrothermal conditions in these regions were generally suitable for forage growth, resulting in high productivity and high WUE (Fig. 1c-d) [52].

### Effect of grazing

From 1979 to 2018, grazing generally decreased CUE in QTPG, indicating that grazing led to a decrease in the proportion of fixed carbon and an increase in the proportion of consumed carbon. The difference in CUE between the grazing and no grazing scenarios increased due to the increase in grazing years and intensity. After grazing, the proportion of immature and vigorously growing tissues in the forage increased [53]. The respiration rate of these new tissues was high, and respiration often accelerated when forage tissue was damaged due to livestock grazing. These factors may explain the overall decrease in CUE caused by grazing in QTPG. However, the impact of grazing on the physiological activities of herbs is very complex. Thus, the impact of grazing on CUE was spatially heterogeneous. The areas where grazing led to a decrease in CUE were predominantly found in the regions where grassland productivity and grazing intensity were both relatively high. In contrast, the areas where grazing led to an increase in CUE were predominantly found in the regions where grassland productivity and grazing intensity were both relatively low (Fig. 1b) [52]. The results of this study suggested that the impact of grazing on CUE is related to the physiological activities of herbs in different natural environments and their responses to different grazing management practices.

From 1979 to 2018, grazing generally decreased WUE in QTPG, and the difference in WUE between the grazing and no grazing scenarios exhibited an increasing trend. Continuous grazing generally led to a decrease in GPP and an increase in ET [49, 54]; thus, the difference in WUE increased. The effect of grazing on WUE has obvious spatial heterogeneity due to differences in the natural environment and grazing management in QTPG (Fig. 1). The regions where grazing obviously decreased WUE were predominantly found in the east and south, which have relatively high grazing intensities. In contrast, the impact of grazing on WUE was relatively weak in the northwest, which has a low grazing intensity.

### Uncertainty

The current study validated the accuracy of the Biome-BGCMuSo model for estimating CUE and WUE in QTPG via comparisons with observational data. However, uncertainty still exists in the results, which is inevitable for any model simulation [25].

First, all models are simplified versions of reality and cannot fully describe complex reality. Thus, complex ecological processes in QTPG are not fully considered in the Biome-BGCMuSo model. For instance, previous studies suggested that the freezing-thawing process of frozen soil on the QTP has a substantial impact on water and carbon storages and fluxes within ecosystems [55]. However, this process has not been well quantified; thus, it has not been well considered in the model [28]. This approach undoubtedly introduced uncertainty into the simulated results. Second, the accuracy of the model inputs strongly affects the simulation results. The most important inputs in the present study were climate and grazing data. Climate data from CMFD were proven to be the most accurate reanalysis data. However, their accuracy was still lower than that of the observation data, which inevitably introduced uncertainty into the simulation results [31]. Grazing data from GLW were developed to provide a statistically informed estimate of how livestock were distributed within a given census unit. To further ensure high accuracy, we used livestock statistics from local governments to correct the data. Nevertheless, use of grazing data still causes uncertainty in the simulation results.

### Comparison with other studies

To date, estimates of grassland CUE and WUE over large areas have attracted increasing amounts of attention [56]. For example, Y Liu, Y Yang, Q Wang, X Du, J Li, C Gang, W Zhou and Z Wang [57] evaluated CUE for global grasslands over the period 2000–2013 using remote sensing data. X Liu, Q Lai, S Yin, Y Bao, S Qing, S Bayarsaikhan, L Bu, L Mei, Z Li, J Niu, et al. [58] explored the spatiotemporal patterns of WUE in Mongolian Plateau grasslands using multisource remote sensing data. These studies deepened our understanding of grassland CUE and WUE. However, previous research has focused on the impact of climate change on CUE and WUE, particularly in grasslands of alpine areas. X Luo, B Jia and X Lai [20] described spatiotemporal changes in CUE across the QTP using 12 terrestrial ecosystem models. S Lin, G Wang, Z Hu, K Huang, J Sun and X Sun [19] estimated the spatiotemporal patterns of WUE on the Tibetan Plateau from 1979 to 2010 using a land surface model. These two studies have provided insights into CUE and WUE on the Tibetan Plateau, but the results are incomplete for grassland CUE and WUE. The studies lacked analysis of the spatiotemporal variations in CUE and WUE in QTPG. W Liu, X Mo, S Liu, Z Lin and C Lv [23] investigated the spatiotemporal variation in WUE in alpine grasslands on the QTP during 2001–2017 using the VIP distributed ecohydrological model. Their study revealed that the yearly mean WUE of the grassland was 0.64 gC/kgH_2_O. N Ma and Y Zhang [24] also investigated the change in WUE of alpine grasslands on the QTP between 1982 and 2016 using a coupled carbon–water model. They found that the average annual WUE was 0.55 gC/kgH_2_O. Both of abovementioned studies suggested that the average annual WUE decreased from the southeastern to the northwestern QTP grasslands. In the present study, the average annual WUE was 0.5591 gC/kgH_2_O from 1979 to 2018 across the QTP grasslands, which was closer to that reported in the study by N Ma and Y Zhang [24]. In addition, WUE generally decreased from the southeast to the northwest across the QTPG, which was similar to the findings of W Liu, X Mo, S Liu, Z Lin and C Lv [23] and Ma and Zhang [24]. Unlike previous studies, we further studied the effect of grazing on spatiotemporal dynamics of WUE across the QTPG. This is the first systematic report on the effect of grazing on WUE over large areas in alpine grasslands. In particular, this is the first specific and systematic investigation of the effect of grazing on CUE across large areas. In the present study, CUE and WUE under both grazing and no grazing scenarios were estimated using the same Biome-BGCMuSo method, which is a functionally holistic approach and avoids the inherent uncertainties stemming from the use of different methods or data sources. The results of the present study deepened our understanding of CUE and WUE in alpine grassland ecosystems and are conducive to the sustainable utilization of grassland resources on the QTP.

## Conclusion

This study estimated and analyzed the spatiotemporal changes in CUE and WUE and their responses to grazing on the QTPG from 1979 to 2018 by utilizing the enhanced Biome-BGCMuSo model. The CUE of the QTPG generally exhibited a fluctuating decreasing trend from 1979 to 2018 due to global warming. A greater percentage of low CUE values occurred in areas with relatively low elevations and relatively high temperatures. A greater percentage of high CUE values occurred at relatively high elevations and relatively low temperatures. Grazing generally led to a decrease in fixed carbon and an increase in consumed carbon in QTPG from 1979 to 2018. The difference in CUE between the grazing and no grazing scenarios increased due to the increase in grazing duration and intensity. The areas where grazing led to a decrease in CUE were predominantly found in the regions with relatively high grassland productivity and grazing intensity. In contrast, the areas where grazing led to an increase in CUE were predominantly found in the regions with relatively low grassland productivity and grazing intensity. After 2000, the grassland WUE on the QTP generally exhibited a fluctuating increasing trend, mainly due to the increase in GPP under the actual climate scenario. Low WUE values were predominantly found in the northwest due to climate and environmental conditions being unsuitable for forage growth. High average annual WUE values were predominantly found in the eastern and southern parts of the QTP because hydrothermal conditions are relatively suitable for forage growth. From 1979 to 2018, grazing generally decreased WUE in QTPG, and the difference in WUE between the grazing and no grazing scenarios exhibited an increasing trend because continuous grazing generally led to a decrease in GPP and an increase in ET. The areas where grazing obviously decreased WUE were predominantly found in the eastern and southern regions, which have relatively high grazing intensity. In contrast, the impact of grazing on WUE was relatively weak in the northwest, which has a low grazing intensity. This study deepens the understanding of CUE and WUE in alpine grassland ecosystems and provides data that could be used to facilitate the sustainable utilization of grassland resources on the QTP.

## Data Availability

The datasets used and/or analyzed during the current study are available from the corresponding author upon reasonable request.

## Abbreviations

CUE: Carbon use efficiency
WUE: Water use efficiency
QTP: Qinghai–Tibet Plateau
QTPG: Qinghai–Tibet Plateau grasslands
ET: Evapotranspiration
NPP: Net primary productivity
GPP: Gross primary productivity
CMFD: China Meteorological Forcing Dataset
FAO: United Nations Food and Agriculture Organization
GLW: Gridded Livestock of the World
